# Liver Damage and Impaired Coagulation in COVID-19 Patients: A Case Series

**DOI:** 10.3390/diseases11040141

**Published:** 2023-10-13

**Authors:** Ludovico Abenavoli, Isabella Aquila, Matteo Antonio Sacco, Giuseppe Guido Maria Scarlata, Anna Caterina Procopio, Luigi Boccuto, Emidio Scarpellini, Marta Greco, Daniela Patrizia Foti, Pietrantonio Ricci, Francesco Luzza

**Affiliations:** 1 Department of Health Sciences, University “Magna Græcia”, Viale Europa, 88100 Catanzaro, Italy; giuseppeguidomaria.scarlata@unicz.it (G.G.M.S.); procopioannacaterina@unicz.it (A.C.P.); marta.greco@unicz.it (M.G.); luzza@unicz.it (F.L.); 2 Institute of Legal Medicine, Department of Medical and Surgical Sciences, University “Magna Graecia”, Viale Europa, 88100 Catanzaro, Italy; isabella.aquila@unicz.it (I.A.); matteo.sacco@unicz.it (M.A.S.); ricci@unicz.it (P.R.); 3 Healthcare Genetics and Genomics Doctoral Program, School of Nursing, College of Behavioral, Social and Health Sciences, Clemson University, Clemson, SC 29631, USA; lboccut@clemson.edu; 4 Translationeel Onderzoek van Gastroenterologische Aandoeningen (T.A.R.G.I.D.), Gasthuisberg University Hospital, KU Leuven, Herestraat 49, 3000 Leuven, Belgium; emidio.scarpellini@med.kuleuven.be; 5 Department of Experimental and Clinical Medicine, University “Magna Græcia”, Viale Europa, 88100 Catanzaro, Italy; foti@unicz.it

**Keywords:** SARS-CoV-2, histology, liver disease, inflammation, vascular disease

## Abstract

The severe acute respiratory syndrome coronavirus 2 (SARS-CoV-2) pandemic has generated an unprecedented challenge for healthcare systems worldwide. Currently, the scientific community wonders if liver injury in patients suffering from severe forms is a direct consequence of the virus or secondary manifestations of systemic inflammation. The liver plays an essential role in the development of the inflammatory storm typical of this disease, and its involvement is associated with worse clinical outcomes and a higher risk of morbidity and mortality from Coronavirus disease 2019 (COVID-19). Methods: Ten patients suffering from severe COVID-19 disease who died between January 2020 and December 2021 were included in the present analysis. These subjects underwent a post mortem examination with a focused evaluation of the hepatic injury. Also, several laboratory parameters have been evaluated, with a primary focus on prothrombin time, partial thromboplastin time, fibrinogen, antithrombin III, and D-dimers to detect coagulative changes. Results: The main cause of death was represented by pulmonary thromboembolism events (50%). The analysis of coagulation laboratory parameters and liver biomarkers revealed a statistically significant rise in aPTT and ALP, and a decrease in albumin, when comparing the blood value at admission and death. We also found high levels of D-dimers in most of the subjects at the time of hospitalization. Interestingly, the post mortem analysis of the liver showed ample morphologic variability, with several disease features. In detail, the liver histology revealed the following: the presence of a variable degree of micro- and macrovacuolar steatosis, inflammation (also, hepato-cholangitis), and variable fibrosis. Of mention, we were also able to detect organized fibrinous material. Conclusions: Our results indicate that in subjects with a severe form of COVID-19, liver disease is related to changes in coagulative and fibrinolytic pathways. In particular, we noted low fibrinogen levels and high D-dimer levels with histological liver findings. Our data suggest that fibrinogen and D-dimers may be used as prognostic markers to detect the severity of liver disease in patients with COVID-19. Finally, we underline the crucial role of coagulation balance in subjects with severe forms of COVID-19.

## 1. Introduction

Severe Acute Respiratory Syndrome Coronavirus 2 (SARS-CoV-2) represents the causal agent of coronavirus disease-2019 (COVID-19) [1,2,3,4]. The COVID-19 pandemic has been characterized by unprecedented social and economic impact. Coronaviruses belong to the Coronaviridae family, a particularly heterogeneous family of positive-sense single-stranded RNA viruses [5,6]. The SARS-CoV-2 genome consists of approximately 29,900 nucleotides arranged in fourteen open reading frames (ORFs) encoding thirty-one proteins; sixteen non-structural proteins involved in genome replication; four structural proteins: spike (S), envelope (E), membrane (M), and nucleocapsid (N); and eleven accessory proteins [7]. These viruses are responsible for infections in humans, mammals, avian species, livestock, and companion animals.

The SARS-CoV-2 pandemic represented the third zoonotic introduction of a coronavirus into humans, preceded by the Severe Acute Respiratory Syndrome Coronavirus (SARS-CoV) and Middle East Respiratory Syndrome Coronavirus (MERS-CoV) epidemics [8,9]. COVID-19 is responsible for a multisystem disease characterized by the presence of a cytokine storm [10,11,12,13]. During COVID-19, the generalized inflammatory state determines a multi-organ involvement with a synergistic effect in the development of the damage connected to the disease. A protein spike interacting with the angiotensin-converting enzyme-2 (ACE-2) penetrates inside the body cells. This receptor is present in various organs and systems including the respiratory, cardiovascular, urogenital, and nervous systems, and is also highly expressed in the gastrointestinal tract, liver, and gallbladder [14]. In this context, understanding the mechanisms of organ damage in districts that are usually not considered is of crucial importance. Valid support in the diagnostic and etiopathogenetic definition of organ damage is provided by laboratory tests, imaging techniques, and post-mortem histological examinations [15,16,17]. In particular, the high presence of ACE-2 receptors in the various anatomical districts is associated, above all, with the severe forms of SARS-CoV-2 infection. Liver damage resembles one of the other clinical features of COVID-19, despite the typical respiratory signs and symptoms [18].

The exact pathophysiology of liver injury in patients with COVID-19 has yet to be elucidated [19,20,21]. Currently, the scientific community discusses whether the liver damage is directly attributable to the viral agent or whether the observed changes are secondary manifestations of the systemic inflammation triggered by SARS-CoV-2. In this regard, the Chinese Digestion Association of Medical Doctor Association and the Chinese Society of Hepatology of Medical Association stated that the development of liver lesions in COVID-19 patients could be related to several pathogenetic mechanisms including damage induced directly by the virus, systemic inflammatory response syndrome, hepatic ischemia and hypoxia, pre-existing liver disease, and drug-induced liver injury [22]. Signs of liver involvement in COVID-19 can be expressed in a severity range from the mild elevation of transaminase and bilirubin levels to severe hepatic dysfunction [23]. Liver involvement is associated with worse clinical outcomes and a higher risk of COVID-19-related morbidity and mortality [24]. Although the importance of the liver in the synthesis of coagulation factors and acute phase proteins is known, the relationship between coagulopathy from COVID-19 and liver damage remains to be clarified. In this frame, it is emerging how the lesions of the liver parenchyma and the imbalance of the coagulation pathway can lead to death in subjects suffering from severe forms of COVID-19. However, limited data are available on coagulation disorders and vascular diseases in patients with COVID-19; the lack of evidence particularly affects findings from liver histology. Recently, it has been suggested that a higher D-dimer level is independently associated with hepatocellular necrosis and suggests that the coagulopathy might be associated with liver damage through microvascular thrombosis associated with systemic inflammation [24]. Reports in the literature describe specific liver histopathological findings, such as the derangement of intra-hepatic blood vessels, venous outflow obstruction, and early organizing thrombi in the portal and terminal hepatic venules [25,26].

We here report the findings from a comprehensive series of patients who died as consequence of severe COVID-19 and underwent post mortem examination; a specific focus was dedicated to histopathological hepatic findings. The clinical and histopathological data collected in this study are discussed in comparison with available data from the scientific literature, with the aim to increase knowledge on the possible pathogenetic mechanisms involved.

## 2. Materials and Methods

The present monocentric case series was performed at the “Magna Graecia” University of Catanzaro (Italy). The analysis included 10 patients (7 males and 3 females) affected by a severe form of COVID-19 and deceased between January 2020 and December 2021, on whom medico-legal examination was performed within 24 h post mortem. All the autopsies were carried out based on the recommendations reported in the official document published by the Italian Ministry of Health, the 1 April 2020 [27]. Histological examination was conducted as follows: liver biopsies were first fixed in 10% neutral buffered formalin for 24 h and histoprocessed. The blocks were then cut into different sections using a rotary microtome. The sections were stained with hematoxylin and eosin stains. Patients’ demographics, the average number of days of hospitalization, comorbidities, the available laboratory data, and cause of death were also recorded. All the post mortem procedures were performed in patients previously hospitalized in intensive care areas.

The infection was confirmed in all cases using real-time polymerase chain reaction (RT-PCR) testing targeting the SARS-CoV-2 genome (Xpert^®^ Xpress SARS-CoV-2, Cepheid, Sunnyvale, CA, USA). Clinical information and laboratory data were acquired at our hospital information system and retrospectively analyzed. The study protocol was approved by the local Research Ethics Committee (n.221/2020). Through the anamnestic evaluation of the subjects’ medical records, it emerged that none of the subjects showed pre-existing or co-existing liver pathologies. Also, all the patients are described as normal weight in the clinical diaries. 

During the hospitalization, the following laboratory parameters were collected for all the patients at hospital admission (T_0_) and death (T_1_): international normalized ratio (INR), activated partial thromboplastin time (aPTT), fibrinogen, antithrombin III (AT-III), D-dimers, aspartate aminotransferase (AST), alanine aminotransferase (ALT), gamma-glutamyl transferase (GGT), alkaline phosphatase (ALP), and albumin. Data were analyzed using a student’s *t*-test for parametric data and a Wilcoxon test for non-parametric data. We assumed a statistical significance for a *p*-value less than 0.05. Furthermore, the study reports the liver histological findings of the subjects enrolled in order to identify the presence of steatosis, inflammation, and fibrosis. 

## 3. Results

The mean patient age was 64 ± 19.11 years, while the mean number of hospitalization days before death was 11.3 ± 9.61 days (Table 1). Considering the emergency condition, we tried to record comorbidities history in our patients. In particular, we reported in patient #3 a positive medical history for heart failure; #4 pulmonary fibrosis; #6 and #8 arterial hypertension; #7 diabetes mellitus type 2 (DM2); and #9 stroke. The patients were treated for COVID-19 in accordance with the Italian national guidelines in force at that time, by using different treatment approaches (oxygen support, steroids, antibiotics) [28]. However, establishing the exact previous therapies prescribed in these patients was not possible.

The subjects examined in the present study, except for patients #7 and #9, were undergoing anticoagulant therapy with low molecular weight heparin (LMWH; SC 50 IU/kg twice daily on the ward, 75 IU/kg twice daily in intensive care). In our cohort, the main cause of death was represented by pulmonary thromboembolism events. In particular, 40% of the subjects died from pulmonary embolism, 30% from disseminated intravascular coagulation, 20% from pneumonia, and 10% from cerebrovascular accident.

Table 2 reports the results of the statistical analysis of the recorded coagulation laboratory parameters and liver biomarkers. The analysis showed a statistical significance result for the aPTT, ALP, and albumin levels, comparing the values at admission (T_0_) vs. the death (T_1_). 

**Figure 1:**
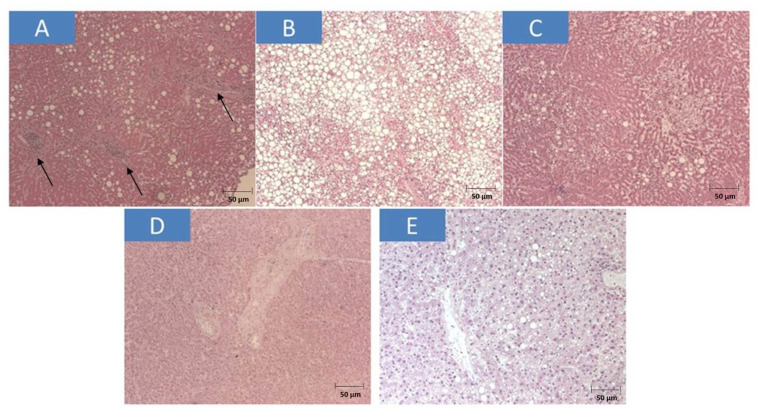
Post mortem liver biopsy specimens. All images are hematoxylin and eosin (H&E magnification)-stained formalin-fixed, paraffin-embedded tissue sections. (**A**) case #6 ×40 (black arrows point to the areas of necrosis), Scale bar = 50 µm; (**B**) case #7 ×100, Scale bar = 50 µm; (**C**) case #8 ×40, Scale bar = 50 µm (**D**) case #9 ×40, Scale bar = 50 µm; (**E**) case #10 ×100, Scale bar = 50 µm, respectively.

Liver macroscopic evaluation during post mortem examination showed the presence of steatosis in the fresh sections of the hepatic tissues studied, with a softening of the parenchyma and visceral congestion. In detail, the histology of liver parenchyma revealed the following: patient #1: macrovescicular steatosis (<10%) and mild chronic inflammation of the portal spaces and mononuclear infiltrate, mainly composed by monocytes and lymphocytes, as a consequence of inflammatory status and vascular congestion; #2: mild steatosis, inflammation and initial fibrosis, loss of cytoarchitectural details probably related to post mortem process of cellular death; #3: diffuse areas of rarefied central lobularity, loss of consistency, regressive hepatocyte aspects and cholestasis, with fibrous expansion and short septa; #4: limited presence of sinusoidal dilatation especially in the central lobe area, mild chronic inflammation, moderate fibrosis; #5: mild macrovescicular steatosis, and blood congestion and hepatocellular cytoplasmic rarefaction, especially at centrolobular level, and also notes of inflammation in portal spaces and cholestasis; #6: micro-mediovacuolar steato-necrosis and hepato-cholangitis, with focal areas of necrosis (Figure 1A); #7: severe micro–macro vacuolar steatosis, with liver parenchyma almost completely stuffing to adipocytes vacuoles (Figure 1B); #8: micro–macrovacuolar steatosis with massive periportal fibrosis associated to inflammatory lymphocytes infiltrated (Figure 1C); #9: chronic hepato-cholangitis with moderate autolysis events and loss of cellular nuclei, in areas of inflammation (Figure 1D); #10: microvacuolar steatosis and diffuse parenchymal vessels congestion (Figure 1E). 

Microscopic examinations of the thrombi detected the presence of organized blood-fibrinous material, associated with granulocytic inflammatory infiltrate.

## 4. Discussion

COVID-19 is a multi-organ disease capable of triggering a systemic response. The literature reports that a SARS-CoV-2 infection can induce hypercoagulability, hypo fibrinolysis, and platelet hyper-activation, in an immuno-thrombo-inflammatory context. Also, increases in both prothrombin time and D-dimer levels are typical laboratory findings in patients with a severe stage of virus infection. This condition has been described as a COVID-19-associated coagulopathy [26].

These phenomena present a relevant clinical impact: vascular atherothrombosis, endothelial damage, and prothrombotic dysregulation of hemostasis in several anatomic districts, including the liver. At the same time, COVID-19-induced liver damage leads to an increase in liver enzymes such as AST, ALT, and ALP, and a decrease in circulating levels of albumin [26,29]. For this reason, hepatic injury has been related to the disease’s progression and severity [30]. However, limited data—especially histological ones—are currently available. There is evidence correlating COVID-19 to Non-Alcoholic Fatty Liver Disease (NAFLD) via pathogenetic mechanisms including cytokine storm and additional multifactorial events [31,32]. In addition, clinical data suggest a prothrombotic state in patients with NAFLD due to its metabolic characteristics, although the complex biochemical pathways involved are not yet fully understood [33]. Some of our patients may have NAFLD, especially those who have DM2. However, this finding does not show up in medical history that was conducted in the emergency setting. Patients’ comorbidities should be considered as some studies show alteration in liver architecture correlated to some concomitant diseases. For example, pathological changes in the liver in heart failure include sinusoidal dilatation and congestion progressing to fibrosis [34] and, as reported previously, there may be hepatic steatosis in patients with DM2 [35]. 

Tsutsumi et al. retrospectively examined a cohort of 60 patients hospitalized with COVID-19 and found in a multivariable analysis that D-dimers are an independent factor for liver dysfunction. It is plausible to infer that the pathogenic mechanism of this coagulopathy might be related to microvascular thrombosis in addition to systemic inflammation [24].

A post mortem study performed by Sonzogni et al. has analyzed liver biopsy samples in 48 patients deceased from severe COVID-19 and described vascular abnormalities such as luminal dilation, and sinusoidal and portal venous micro-thromboses in almost all subjects [25]. Also, portal inflammation, portal fibrosis, and micro- and macro-vesicular steatosis were shown. The possible explanation of these results is that endothelial injury or coagulation dysfunction might be the primus movens of COVID-19 liver damage.

An unrelated study on COVID-19 patients with acute respiratory failure found a severe hypercoagulability characterized by significantly higher levels of both fibrinogen and D-dimers, associated with a marked hyper-coagulable thrombo-profile [36]. This observation reflects a severe form of hypercoagulability, rather than a consumption coagulopathy. Rampotas and Pavord compared blood films from COVID-19 patients under invasive ventilation and non-severe COVID-19 cases, respectively. The presence of platelet aggregates and macro-thrombocytes was found in the first group, indicating increased platelet activity [37]. These morphology findings could be consistent and underline the central role played by platelets in the development of thrombotic complications during severe COVID-19 infection. 

Fanni et al. utilized histology and scanning electron microscopy to analyze liver biopsies from four patients with COVID-19 and described mild inflammation, sinusoidal changes with dilatation, thrombosis, and diffuse fibrin deposition [38]. These pathological changes suggested that the vascular involvement with intra-sinusoidal thrombosis could be the typical feature of SARS-CoV-2-related liver injury.

In a prospective study of 267 newly diagnosed COVID-19 patients, Esmaeel et al. reported that a marked hypercoagulability state characterized by high aPTT blood levels is more frequently found in non-survival subjects compared with patients with non-severe forms [39].

Considering this background, in our cohort we found seven patients who died from thromboembolic events. In particular, three subjects passed away because of pulmonary embolism, one due to a cerebrovascular accident, and three because of disseminated intravascular coagulation. These data confirm the presence of an unbalanced coagulation profile in patients with severe forms of COVID-19.

The described features found when analyzing the liver damage in COVID-19 resemble those typical of advanced liver disease, namely cirrhosis, or liver failure. In detail, in our study, the analyses of aPTT values upon admission showed that all the examined patients presented values below normal, suggesting a high risk of thrombotic events. However, at the time of death, only two patients presented values greater than 110 s, and two presented aPTT values at the low reference value. Thus, at the moment of death, they looked like cirrhotic patients. Indeed, patient #1, who presented an aPTT of 115 s, and patient #6, who presented an aPTT of 121 s, both died due to thromboembolism, a condition related to severe consumption coagulopathy, despite a “cirrhosis-like” anti-thrombotic state at blood tests. Therefore, this element is compatible with a picture of systemic hypercoagulability confirmed by the finding of hepatic micro-thromboembolism at autopsy and histological investigations. Hepatic micro-thromboembolism was also detected in cases with deranged liver cirrhosis that have a higher procoagulant tendency within the portal and sinusoidal veins but a high bleeding risk outside of the liver.

Interestingly, eight patients presented aPTT values persistently below the cut-off values at exitus. This observation is traced back to the robust systemic inflammation observed in subjects with severe forms of COVID-19, characterized by disseminated intravascular coagulation. Fibrinogen is an acute-phase protein synthesized by the liver during inflammatory states. In this regard, the highest fibrinogen values were found in subjects #1, #5, and #7 who died, respectively, of thromboembolic disease, cerebrovascular accident, and disseminated intravascular coagulation. On the contrary, low fibrinogen levels were observed in patients #3, #4, and #6 which could be associated with a direct viral liver injury, resulting in impaired coagulation synthesis. In this context, we must recognize the multifactorial liver damage caused by SARS-CoV-2 that is explained by a direct viral effect on hepatocytes and/or biliary tract cells, inflammatory response within hepatic tissue, hypoxic damage, and drug-related causes [2]. These fibrinogen values confirm both the systemic and generalized inflammation typical of cytokines’ storm for COVID-19 and suggest a reduced synthesis, perhaps associated with direct and indirect liver damage. 

The increased blood values of D-dimers indicate a disequilibrium between coagulation and fibrinolysis pathways [36,37]. In this regard, the study showed high levels of D-dimers in most of the subjects at the time of hospitalization. This parameter was particularly altered in the case of patients #2 and #5 at the time of hospital admission. Furthermore, the study showed that the D-dimer values underwent a marked increase during the patient’s hospitalization, reaching limits well beyond the maximum threshold foreseen. This condition is strongly associated with phenomena of pulmonary embolism, disseminated intravascular coagulation conditions that have represented the main causes of death in the subjects described. 

In conclusion, the coagulation parameters measured in our cohort resemble the trends shown by these “inflammatory biomarkers”. In fact, the latter can fall within healthy levels and be up- or down-regulated. Importantly, when levels of von Willebrand factor (VWF), P-selectin, and fibrinogen increase, with a normal or slightly increased D-dimer concentration, a rapid, aggressive COVID-19 progression must be acknowledged. This may be associated with thrombocytopenia and bleeding risk. Further, subsequent VWF and fibrinogen depletion and a rise in D-dimer levels are generally followed by the cytokine storm outbreak, which is suggestive of poor prognosis [40]. The results of our study revealed only a rise in prothrombin time and a tendency for D-dimer concentration. This is partially in line with evidence from the literature. Data on potential thrombocytopenia are lacking. Indeed, our sample is variegated and describes different features of fatal COVID-19 disease where the causes of exitus are not only related to thrombosis. Finally, these various coagulation derangements can be found also at the histological level and can also be conditioned by the presence of liver involvement, namely steatosis, fibrosis, and micro-inflammation [41].

Autoptic examination represents the gold standard to study the exact cause of death in all subjects and especially in those with COVID-19. A post mortem examination could provide information on the pathogenesis and pathophysiology of the SARS-CoV-2 infection, with potential therapeutic implications. The patients included in our report presented hepatic damage on post mortem gross and histologic examinations. This finding can be considered a consequence of systemic damage related to COVID-19. In fact, none of them had pre-existing hepatic disease justifying the liver damage found at the histologic examination carried out during autopsy. 

More interestingly, the results of this investigation are consistent with the data currently available in the scientific literature. Indeed, in addition to the increase in aminotransferase values, circulating ALP levels were significantly increased between hospital admission and patient death. First, a study conducted by Xu Z. et al. demonstrated that SARS-CoV-2 infection can induce liver steatosis [42]. Moreover, a worse COVID-19 and hepatic failure can be predicted by rising aminotransferase values and initial ALP increase upon admission. A recent study showed that at the time of admission, the percentage of patients with elevated ALP in severe cases was 18.6% and was significantly higher than 14.6% in patients with mild disease [43]. The explanation for the increase in this biomarker is the SARS-CoV-2 binding to the ACE-2 receptor also expressed in the bile ducts. Such binding induces cholangiocytes damage and subsequent cholestasis [44]. In agreement with the current literature, circulating levels of albumin in patients under investigation showed a significant decrease between hospital admission and exitus. Indeed, a recent study conducted by Xu Y. et al. showed that albumin levels were reduced in patients with COVID-19 and that hypoalbuminemia was a predictor of adverse prognosis [45]. This evidence was confirmed by a multicenter retrospective observational study conducted on 296 COVID-19 patients attending three different Italian hospitals [46]. Albumin—produced by the liver—is the most abundant protein in human serum and it functions as an antioxidant. The hypoalbuminemia detected in our patients could be explained because the inflammation caused by COVID-19 involves a degradation of circulating albumin. At the same time, COVID-19-related liver damage impairs albumin synthesis, making an imbalance between its synthesis and degradation [47].

These features are shared by subjects with pre-existing liver disease and individuals with de novo hepatic damage. However, none of the patients enrolled in this study had pre-existing liver disease. Moreover, patients with liver steatosis and, especially, steatohepatitis are more prone to liver damage, failure progression, and, lately, fatal COVID-19 prognosis. Finally, acute respiratory distress syndrome damages liver cells through the induction of extensive hypoxia. This mechanistic process can also explain the rapid increase in liver laboratory test values observed in this subset of patients and, specifically, in our cohort of patients, perhaps with a fatal COVID-19 course. Other than inflammatory cells found in liver specimens from our patients, there are also those with signs of fibrosis. In this regard, an aberrant immune response involves Kupffer cells activating stellate cells with collagen sediments and fibrosis [39]. This rapid spreading of liver fibrosis, typical of severe cases of COVID-19 patients with concomitant liver failure, is consistent with the rapid fibrotic evolution of pulmonary disease in these subjects. 

The present study had several limitations. First, the partially retrospective experimental design has been influenced by the evolution of the pandemic, leading, in some instances, to an incomplete collection of clinical records, including a history of liver disease, alcohol use, and other risk factors. Secondly, only a limited sample size was available for real-world data, especially in emergency conditions. Also, any potentially confounding bias related to the impact of drug therapy on liver markers could not be assessed because of the rapidly changing clinical course of COVID-19 in several patients. The latter could be overcome in future multicenter investigations focusing on larger cohorts.

## 5. Conclusions

The high number of deaths associated with COVID-19 and the lack of knowledge of the medium- and long-term outcomes require a careful consideration of the pathogenetic mechanisms behind it. Laboratory tests and post mortem histological examinations are fundamental diagnostic and pathophysiologic tools for understanding this disease. Unfortunately, the terrible impact of the pandemic on our healthcare system has hampered the sharing of laboratory and autoptic data among researchers. Thus, despite considerable ongoing efforts, the mechanisms of COVID-19 hepatic impairment remain to be elucidated. Hypercoagulability, hypofibrinolysis, platelet count, and functioning changes are physiopathological features of severe forms of SARS-CoV-2 infection, related to the onset and development of an immuno-thrombo-inflammation clinical picture. These features are clinically relevant, resulting in manifestations of thrombosis in a variety of anatomic districts. Our data support the concept that liver dysfunction could be due to microvascular portal thrombosis. Although preliminary and partial, these observations would help the effective clinical and therapeutic management of COVID-19 patients associated with coagulation disorders and liver injury.

## Figures and Tables

**Table 1:** Clinical features, coagulative profile, and liver biomarkers at hospital admission (T_0_) and death (T_1_).

Patients	#1	#2	#3	#4	#5	#6	#7	#8	#9	#10
Age	51	52	85	74	77	62	64	71	83	21
Sex	M	M	M	M	F	M	F	M	F	M
Hospital stay (days)	9	18	27	12	10	26	7	2	1	1
Comorbidities	-	-	HF	PF	-	AH	DM2	AH	Stroke	-
Cause of death	PE	PNA	PNA	PE	CVA	PE	DIC	PE	DIC	DIC
INR										
T_0_	1.06	0.96	0.89	1.22	3.57	0.91	1	1.13	1.3	1.42
T_1_	1.03	1.17	3.03	1.33	1.28	1.77	1.13	1.2	1.37	1.45
aPTT (s)										
T_0_	31	15	35	39	37	33	35	58	39	31
T_1_	115	40	67	39	40	121	40	67	40	35
Fibrinogen (mg/dL)										
T_0_	546	164	368	303	618	603	636	542	448	604
T_1_	960	500	220	182	812	585	708	563	453	608
AT-III (%)										
T_0_	80	120	85	92	97	106	100	84	95	100
T_1_	71	118	110	106	82	73	80	88	97	111
D-Dimers (μg/L)										
T_0_	0.77	15.38	0.75	0.44	5.89	0.38	0.95	1.13	1.89	0.79
T_1_	6.83	15.38	0.75	0.38	5.89	4.44	3.67	1.13	1.86	0.81
AST (UI/L)										
T_0_	45	27	25	18	15	24	27	27	38	19
T_1_	92	63	1886	87	641	24	30	23	56	13
ALT (UI/L)										
T_0_	62	62	12	19	19	19	38	51	54	12
T_1_	52	36	162	23	469	18	40	53	50	25
GGT (UI/L)										
T_0_	21	68	21	28	69	38	53	91	34	30
T_1_	39	271	96	14	151	13	58	73	47	45
ALP (UI/L)										
T_0_	46	74	80	84	62	63	67	42	81	75
T_1_	180	261	103	55	128	72	105	30	101	100
Albumin (g/dL)										
T_0_	3.2	3.3	3.7	3.2	3.0	3.9	3.5	3.4	3.8	3.1
T_1_	2	2.6	3.5	3.1	3.2	2.9	3.2	2.8	2.4	2.4

HF: heart failure; PF: pulmonary fibrosis; AH: arterial hypertension; DM2: diabetes mellitus type 2. PE: pulmonary embolism; PNA: pneumonia; CVA: cerebrovascular accident; DIC: disseminated intravascular coagulation; AT-III: Antithrombin III, AST: aspartate aminotransferase, ALT: alanine aminotransferase, GGT: gamma-glutamyl transferase, ALP: Alkaline phosphatase.

**Table 2:** Statistical analysis of the coagulation laboratory parameters and liver biomarkers at admission (T_0_) and death (T_1_).

	T_0_	T_1_	*p*-Value
INR	1.35 ± 0.8	1.48 ± 0.58	0.114
aPTT (s)	35.3 ± 10.54	60.4 ± 32.5	0.009
Fibrinogen (mg/dL)	438.2 ± 158.72	559.1 ± 240.8	0.226
AT-III (%)	95.88 ± 11.76	85.27 ± 32.01	0.689
D-Dimers (μg/L)	2.84 ± 4.7	4.13 ± 4.57	0.052
AST (UI/L)	26.5 ± 9.1	291.5 ± 590.1	0.17
ALT (UI/L)	34.8 ± 20.9	92.8 ± 138.4	0.20
GGT (UI/L)	45.7 ± 23.7	80.7 ± 78.3	0.19
ALP (UI/L)	67.5 ± 14.5	113.5 ± 65.9	0.044
Albumin (g/dL)	3.41 ± 0.3	2.71 ± 0.5	0.0009

## Data Availability

The data presented in this study are available on request from the corresponding author.

## References

[1] Borczuk A.C., Yantiss R.K. (2022). The pathogenesis of coronavirus-19 disease. J. Biomed. Sci..

[2] Montori M., Baroni G.S., Santori P., Di Giampaolo C., Ponziani F., Abenavoli L., Scarpellini E. (2023). Liver Damage and COVID-19: At Least a “Two-Hit” Story in Systematic Review. Curr. Issues Mol. Biol..

[3] Zanza C., Racca F., Longhitano Y., Piccioni A., Franceschi F., Artico M., Abenavoli L., Maiese A., Passaro G., Volonnino G. (2021). Risk Management and Treatment of Coagulation Disorders Related to COVID-19 Infection. Int. J. Environ. Res. Public Health.

[4] Abenavoli L., Cinaglia P., Procopio A.C., Serra R., Aquila I., Zanza C., Longhitano Y., Artico M., Larussa T., Boccuto L. (2021). SARS-CoV-2 Spread Dynamics in Italy: The Calabria Experience. Rev. Recent Clin. Trials.

[5] Cui J., Li F., Shi Z.L. (2019). Origin and evolution of pathogenic coronaviruses. Nat. Rev. Microbiol..

[6] Mousavizadeh L., Ghasemi S. (2021). Genotype and phenotype of COVID-19: Their roles in pathogenesis. J. Microbiol. Immunol. Infect..

[7] Redondo N., Zaldívar-López S., Garrido J.J., Montoya M. (2021). SARS-CoV-2 Accessory Proteins in Viral Pathogenesis: Knowns and Unknowns. Front. Immunol..

[8] Hu B., Guo H., Zhou P., Shi Z.L. (2021). Characteristics of SARS-CoV-2 and COVID-19. Nat. Rev. Microbiol..

[9] V’kovski P., Kratzel A., Steiner S., Stalder H., Thiel V. (2021). Coronavirus biology and replication: Implications for SARS-CoV-2. Nat. Rev. Microbiol..

[10] Roberts M.C., Levi M., McKee M., Schilling R., Lim W.S., Grocott M.P.W. (2020). COVID-19: A complex multisystem disorder. Br. J. Anaesth..

[11] Yang L., Xie X., Tu Z., Fu J., Xu D., Zhou Y. (2021). The signal pathways and treatment of cytokine storm in COVID-19. Signal. Transduct. Target. Ther..

[12] Fajgenbaum D.C., June C.H. (2020). Cytokine Storm. N. Engl. J. Med..

[13] Kulanthaivel S., Kaliberdenko V.B., Balasundaram K., Shterenshis M.V., Scarpellini E., Abenavoli L. (2021). Tocilizumab in SARS-CoV-2 Patients with the Syndrome of Cytokine Storm: A Narrative Review. Rev. Recent Clin. Trials.

[14] Zanza C., Tassi M.F., Romenskaya T., Piccolella F., Abenavoli L., Franceschi F., Piccioni A., Ojetti V., Saviano A., Canonico B. (2021). Lock, Stock and Barrel: Role of Renin-Angiotensin-Aldosterone System in Coronavirus Disease 2019. Cells.

[15] Yüce M., Filiztekin E., Özkaya K.G. (2021). COVID-19 diagnosis—A review of current methods. Biosens. Bioelectron..

[16] Dell’Aquila M., Cattani P., Fantoni M., Marchetti S., Aquila I., Stigliano E., Carbone A., Oliva A., Arena V. (2020). Postmortem Swabs in the Severe Acute Respiratory Syndrome Coronavirus 2 Pandemic: Report on 12 Complete Clinical Autopsy Cases. Arch. Pathol. Lab. Med..

[17] Hanley B., Lucas S.B., Youd E., Swift B., Osborn M. (2020). Autopsy in suspected COVID-19 cases. J. Clin. Pathol..

[18] Méndez-Sánchez N., Valencia-Rodríguez A., Qi X., Yoshida E.M., Romero-Gómez M., George J., Eslam M., Abenavoli L., Xie W., Teschke R. (2020). What Has the COVID-19 Pandemic Taught Us so Far? Addressing the Problem from a Hepatologist’s Perspective. J. Clin. Transl. Hepatol..

[19] Testino G., Pellicano R. (2020). Alcohol consumption in the COVID-19 era. Minerva Gastroenterol. Dietol..

[20] Spearman C.W., Aghemo A., Valenti L., Sonderup M.W. (2021). COVID-19 and the liver: A 2021 update. Liver Int..

[21] Actis G.C., Ribaldone D.G., Fagoonee S., Pellicano R. (2021). COVID-19: A user’s guide, status of the art and an original proposal to terminate viral recurrence. Minerva Med..

[22] Chinese Digestion Association, Chinese Medical Doctor Association, Chinese Society of Hepatology, Chinese Medical Association (2020). The protocol for prevention, diagnosis and treatment of liver injury in coronavirus disease 2019. Zhonghua Gan Zang Bing Za Zhi.

[23] Testino G., Pellicano R. (2021). Acute-on-chronic liver failure by SARS-CoV-2 in active alcohol use disorder cirrhotic patient. Minerva Gastroenterol..

[24] Tsutsumi T., Saito M., Nagai H., Yamamoto S., Ikeuchi K., Lim L.A., Adachi E., Koga M., Okushin K., Akai H. (2021). Association of coagulopathy with liver dysfunction in patients with COVID-19. Hepatol. Res..

[25] Sonzogni A., Previtali G., Seghezzi M., Grazia Alessio M., Gianatti A., Licini L., Morotti D., Zerbi P., Carsana L., Rossi R. (2020). Liver histopathology in severe COVID 19 respiratory failure is suggestive of vascular alterations. Liver Int..

[26] D’Ardes D., Boccatonda A., Cocco G., Fabiani S., Rossi I., Bucci M., Guagnano M.T., Schiavone C., Cipollone F. (2022). Impaired coagulation, liver dysfunction and COVID-19: Discovering an intriguing relationship. World J. Gastroenterol..

[27] Rapporto ISS COVID-19 n. 6/2020—Procedura per L’esecuzione di Riscontri Diagnostici in Pazienti Deceduti Con Infezione da SARS-CoV-2. Versione del 23 Marzo 2020. https://www.iss.it/documents/20126/0/Rapporto+COVID-19+n.+6_2020+Autopsie+v27+marzo.pdf/c4b363a1-a246-c36c-d007-ae24ed7e648b?t=1585307031219.

[28] Mussini C., Falcone M., Nozza S., Sagnelli C., Parrella R., Meschiari M., Petrosillo N., Mastroianni C., Cascio A., Iaria C. (2021). Therapeutic strategies for severe COVID-19: A position paper from the Italian Society of Infectious and Tropical Diseases (SIMIT). Clin. Microbiol. Infect..

[29] Abenavoli L., Aquila I., Sacco M.A., Procopio A.C., Cinaglia P., Zanza C., Longhitano Y., Arena V., Fagoonee S., Ricci P. (2023). Liver injury associated with high value of D-dimer plasmatic level in COVID-19 patients. Minerva Gastroenterol..

[30] Bryce C., Grimes Z., Pujadas E., Ahuja S., Beasley M.B., Albrecht R., Hernandez T., Stock A., Zhao Z., Al Rasheed M.R. (2021). Pathophysiology of SARS-CoV-2: The Mount Sinai COVID-19 autopsy experience. Mod. Pathol..

[31] Miranda C., Garlatti E., Da Porto A., Rinaldo E., Grazioli S., Zanette G., Tonizzo M. (2023). Liver injury in COVID-19 patients with non-alcoholic fatty liver disease: An update. Arch. Med. Sci. Atheroscler. Dis..

[32] Dietrich C.G., Geier A., Merle U. (2023). Non-alcoholic fatty liver disease and COVID-19: Harmless companions or disease intensifier?. World J. Gastroenterol..

[33] Ciavarella A., Gnocchi D., Custodero C., Lenato G.M., Fiore G., Sabbà C., Mazzocca A. (2021). Translational insight into prothrombotic state and hypercoagulation in nonalcoholic fatty liver disease. Thromb. Res..

[34] Louie C.Y., Pham M.X., Daugherty T.J., Kambham N., Higgins J.P. (2015). The liver in heart failure: A biopsy and explant series of the histopathologic and laboratory findings with a particular focus on pre-cardiac transplant evaluation. Mod. Pathol..

[35] Masarone M., Rosato V., Aglitti A., Bucci T., Caruso R., Salvatore T., Sasso F.C., Tripodi M.F., Persico M. (2017). Liver biopsy in type 2 diabetes mellitus: Steatohepatitis represents the sole feature of liver damage. PLoS ONE.

[36] Spiezia L., Boscolo A., Poletto F., Cerruti L., Tiberio I., Campello E., Navalesi P., Simioni P. (2020). COVID-19-Related Severe Hypercoagulability in Patients Admitted to Intensive Care Unit for Acute Respiratory Failure. Thromb. Haemost..

[37] Rampotas A., Pavord S. (2021). Platelet aggregates, a marker of severe COVID-19 disease. J. Clin. Pathol..

[38] Fanni D., Cerrone G., Saba L., Demontis R., Congiu T., Piras M., Gerosa C., Suri J.S., Coni P., Caddori A. (2021). Thrombotic sinusoiditis and local diffuse intrasinusoidal coagulation in the liver of subjects affected by COVID-19: The evidence from histology and scanning electron microscopy. Eur. Rev. Med. Pharmacol. Sci..

[39] Esmaeel H.M., Ahmed H.A., Elbadry M.I., Khalaf A.R., Mohammed N.A., Mahmoud H.A., Taha E.M. (2022). Coagulation parameters abnormalities and their relation to clinical outcomes in hospitalized and severe COVID-19 patients: Prospective study. Sci. Rep..

[40] Grobler C., Maphumulo S.C., Grobbelaar L.M., Bredenkamp J.C., Laubscher G.J., Lourens P.J., Steenkamp J., Kell D.B., Pretorius E. (2020). COVID-19: The Rollercoaster of Fi-brin(Ogen), D-Dimer, Von Willebrand Factor, P-Selectin and Their Interactions with Endothelial Cells, Platelets, and Erythrocytes. Int. J. Mol. Sci..

[41] Medeiros A.K., Barbisan C.C., Cruz I.R., Araújo E.M., Libânio B.B., Albuquerque K.S., Torres U.S. (2020). Higher frequency of hepatic steatosis at CT among COVID-19-positive patients. Abdom. Radiol..

[42] Xu Z., Shi L., Wang Y., Zhang J., Huang L., Zhang C., Liu S., Zhao P., Liu H., Zhu L. (2020). Pathological findings of COVID-19 associated with acute respiratory distress syndrome. Lancet Respir. Med..

[43] Krishnan A., Prichett L., Tao X., Alqahtani S.A., Hamilton J.P., Mezey E., Strauss A.T., Kim A., Potter J.J., Chen P.H. (2022). Abnormal liver chemistries as a predictor of COVID-19 severity and clinical outcomes in hospitalized patients. World J. Gastroenterol..

[44] Da B.L., Suchman K., Roth N., Rizvi A., Vincent M., Trindade A.J., Bernstein D., Satapathy S.K. (2021). Northwell COVID-19 Research Consortium. Cholestatic liver injury in COVID-19 is a rare and distinct entity and is associated with increased mortality. J. Intern. Med..

[45] Xu Y., Yang H., Wang J., Li X., Xue C., Niu C., Liao P. (2021). Serum Albumin Levels are a Predictor of COVID-19 Patient Prognosis: Evidence from a Single Cohort in Chongqing, China. Int. J. Gen. Med..

[46] Turcato G., Zaboli A., Kostic I., Melchioretto B., Ciccariello L., Zaccaria E., Olivato A., Maccagnani A., Pfeifer N., Bonora A. (2022). Severity of SARS-CoV-2 infection and albumin levels recorded at the first emergency department evaluation: A multicentre retrospective observational study. Emerg. Med. J..

[47] Wiedermann C.J. (2021). Hypoalbuminemia as Surrogate and Culprit of Infections. Int. J. Mol. Sci..

